# The Biogenically Efficient Synthesis of Silver Nanoparticles Using the Fungus *Trichoderma harzianum* and Their Antifungal Efficacy against *Sclerotinia sclerotiorum* and *Sclerotium rolfsii*

**DOI:** 10.3390/jof8060597

**Published:** 2022-06-02

**Authors:** Ranya M. S. El-Ashmony, Nouf S. S. Zaghloul, Marija Milošević, Mohamed Mohany, Salim S. Al-Rejaie, Yasmine Abdallah, Anwar A. Galal

**Affiliations:** 1 Department of Plant pathology, Minia University, Elminya 61519, Egypt; ranya.elashmoni@mu.edu.eg (R.M.S.E.-A.); anwar.galal@mu.edu.eg (A.A.G.); 2 Bristol Centre for Functional Nanomaterials, HH Wills Physics Laboratory, Tyndall Avenue, University of Bristol, Bristol BS8 1FD, UK; nz17347@bristol.ac.uk; 3 Department of Biology and Ecology, Faculty of Science, University of Kragujevac, 34000 Kragujevac, Serbia; marija.milosevic@pmf.kg.ac.rs; 4 Department of Pharmacology and Toxicology, College of Pharmacy, King Saud University, Riyadh 11451, Saudi Arabia; mmohany@ksu.edu.sa (M.M.); rejaie@ksu.edu.sa (S.S.A.-R.)

**Keywords:** silver nanoparticles, biogenic, *Trichoderma* extract, antifungal, *Sclerotinia sclerotiorum* and *Sclerotium rolfsii*

## Abstract

Silver nanoparticles (AgNs) are known as a promising alternative tool to control fungal diseases. AgNs were biologically synthesized using *Trichoderma harzianum filtrate* as an ecofriendly approach. The presence of AgNs was confirmed by changing the color to brown, followed by UV-Vis spectroscopy, transmission electron microscopy (TEM), and Energy-dispersive spectra (EDS). TEM studies showed that the size of AgNs average was 31.13 nm and the shape was spherical. In vitro assays of AgNs showed a significant inhibitory effect on the growth of *Sclerotinia sclerotiorum* (*S. sclerotiorum*) and *Sclerotium rolfsii* (*S. rolfsii*). The percentage inhibition on mycelial linear growth, dry weight, and sclerotia formation of *S. sclerotiorum* and *S. rolfsii* at 100^−L^ were 87.8, 82.7, 96.4, 52.8, 55.1, and 85.4%, respectively. The obtained results suggested that the biosynthesized AgNs have antifungal activity against *S. sclerotiorum* and S. *rolfsii*. Foliar spray of bean and sunflower plants with AgNs caused a decrease in disease severity, which promoted the plant protection against *S. sclerotiorum* and *S. rolfsii*, respectively. Substantially, this study will extend our understanding of the AgNs antifungal action for suppressing fungal diseases.

## 1. Introduction

Nanotechnology is a growing discipline of research that has applications in numerous sectors, including health and agriculture [1]. In agriculture, nanotechnology can be exploited by using natural resources in the conservation, production, and protection of crops [2]. Recently, biosynthesis of nanoparticles (NPs) or green synthesis of NPs has received much attention due to the biocompatibility, low toxicity, and eco-friendly nature of the process and its products [3]. The use of fungi as reducing and stabilizing agents in the biogenic synthesis of silver nanoparticles has attracted interest due to the enormous amounts of proteins produced, excellent yields, ease of handling, and low toxicity of the residues [4]. Furthermore, the nanoparticles are coated with numerous biomolecules produced from the fungus during the production process, which might improve their stability and biological activity [5]. Ibrahim et al. [5] showed that several fungus species have the potential to be used in biogenic synthesis, allowing for the generation of nanoparticles with a variety of properties, including size, surface charge, and shape. Silver nanoparticles made from fungi might aid in disease management, with benefits such as minimal toxicity and high biocompatibility [6,7]. The discoveries indicated above might contribute to future research into the use of these nanoparticles as antimicrobials in human and animal health and agriculture [8]. The production of capping from fungal biomolecules, improved stability, and biological activity are all advantages of the biogenic synthesis of silver nanoparticles through fungi [9].

*Trichoderma harzianum* is a mycoparasitic filamentous fungus used as an agent in biological control for combating plant pathogens that affect the production of several agriculturally essential plant species [10]. The primary mechanism of its action is the coiling of hyphae and the release of hydrolytic enzymes that degrade the cell wall of the target fungus [10]. Fungi of the genus *Trichoderma* spp. present the NADH co-enzyme and NADH-dependent enzymes such as nitrate reductase, which is essential in synthesizing both the nanoparticles and the cappings that confer more stability [11,12].

The two phytopathogenic fungi *Sclerotinia* *sclerotiorum* and *Sclerotium rolfsii* are a serious concern for a variety of crops, including tomato, beans, and sunflower, resulting in economic losses in several countries worldwide. Resistant structures (sclerotia) can remain viable in the soil for decades and can be widely distributed, leading to plant diseases that cause annual economic losses [13]. Under favorable conditions, they affect all plant organs, including stems, roots, fruits, petioles, and leaves. Considering these detrimental effects of *S. sclerotiorum* and *S. rolfsii* [14], this study aimed to investigate in vitro activities of the Ag nanoparticles obtained through biogenic synthesis by *T. harizanium* against the phytopathogens and their possible effects on their host plant.

## 2. Materials and Methods

### 2.1. Causal Agent and Bioagent

*Sclerotinia sclerotiorum* isolate SS3 was obtained from bean pods, *Sclerotium rolfsii* isolate Sr1 was obtained from sunflower with basal stem rot and the bioagent *Trichoderma harzianum* isolate No. A1 d (TrA1d) was purchased from the Fitogen Plant Diseases lab. Varsak Zeytinlik, Kepez, Antalya, Turkey [15].

### 2.2. Extracellular Synthesis and Characterization of AgNs

The fungal mycelium grown on potato dextrose agar (PDA) was inoculated into the production medium (potato dextrose broth (PDB)) followed by incubation at 28 °C for 5 days. Fully grown mycelium was washed with sterile distilled water to remove medium components. 5 g of *T. harzianum* fungus wet biomass was added to a 100 mL aqueous solution of 1 mM silver nitrate (AgNO_3_), and the resulting mixture was shaken at 100 rpm for 12 days at 28 °C in the dark until the color shifted from bright yellow to dark brown, indicating that the synthesis was complete. The reduction in metallic silver to silver ions was used to make AgNs [16], by using collected dark brown solution, which was washed three times with distilled H_2_O, and then dried overnight in the oven at 150 °C. The obtained nanoparticles were analyzed by ultraviolet spectra at the wavelength range from 200 to 700 nm (T80 spectrometer, PG Instruments Limited, Woodway Lane, Alma Park, Leicestershire, LE17 5FB, United Kingdom). The nanoparticles were examined by electron microscopy (TEM) using (JEOL JEM-100CX II, Tokyo, Japan). A solution of AgNs was dropped to the grid. Energy dispersion spectrum (EDS) was performed to confirm the presence of the elements.

### 2.3. Antifungal Activity of AgNs against S. sclerotiorum and S. rolfsii

#### 2.3.1. Crude Culture Filtrate (CF)

One plug (5 mm diameter) of *T. harzianum* collected from actively developing margins of PDA cultures was used to inoculate 250 mL Erlenmeyer flasks with 50 mL liquid sterilized potato dextrose broth (PDB). Some stationary cultures were incubated at 25 °C for 15 days. The cultures were vacuum filtered through filter paper, and the filtrates (CF) were kept at 2 °C for 24 h [17].

#### 2.3.2. Effect of AgNs on Some Growth Parameters of *S. sclerotiorum* and *S. rolfsii*

The effect of AgNs were evaluated at selected concentrations (25, 50, and 100 µg/mL), with each concentration added to autoclaved nutritional agar medium in conical flasks and then dispensed in Petri dishes (15 mL media/dish) and allowed to harden. Dishes were then inoculated with *S. sclerotiorum* and *S. rolfsii* by placing cork borer made agar discs (5 mm) taken from the periphery of fungal colonies grown for seven days after incubation at 20 °C for *S. sclerotiorum* and 27 °C for *S. rolfsii* in the center of Petri plates containing various concentrations of AgNs and AgNO_3_. For comparison, dishes with nutrient agar medium without AgNs were inoculated identically. The two diameters of the fungal colony, as well as the points at which the mycelium development approached the dish’s edge, were measured. After 14 days, when the sclerotia had covered the control plates entirely, sclerotia from each plate were collected, and their counts were recorded using a magnifying 40× lens [18].

#### 2.3.3. Effect of AgNs on the Mycelial Dry Weight of *S. sclerotiorum* and *S. rolfsii*

The effect of several AgNs levels on the development of *S.* *sclerotiorum* and *S.* *rolfsii* isolates in liquid nutrient broth (NB) medium were investigated (NB Discs of 5 mm diameter were taken from the active edge of 7-day-old cultures of the tested *S. sclerotiorum* at 20 °C and *S. rolfsii* at 27 °C isolates cultivated on nutrient agar medium and used to inoculate 250 mL Erlenmeyer flasks containing 50 mL autoclaved nutrient medium amended with diverse doses of AgNs. The concentrations were prepared with sterile distilled water, and aliquots were pipetted into an NB medium to generate concentrations of AgNs 25, 50, and 100 g/mL. Conical flasks with medium without AgNs were inoculated in the same way as for the untreated control and served as a control. After being isolated from the fungal biomass by filtering using Whatman No-1 filter paper and dried at 60 °C for 48 h, the mycelial dry weight (MDW) of different treatments was assessed (mg MDW per 50 mL liquid medium). The % suppression of fungal growth was calculated using the algorithm reported previously [19].

#### 2.3.4. Effects of AgNs on Root and Crown Rot Severity Caused by *S. sclerotiorum*

Test solutions of AgNs with 25, 50 and 100 µg/mL were added, the plants were sprayed as mentioned previously and the soils were potted in 20 cm diameter sterilized pots. The soil was mixed with *S. sclerotiorum* infested barley grains (3.0% *w*/*w*); all pots were irrigated regularly and kept under greenhouse conditions. Seven days later, pots were cultivated with seeds of bean cv. Giza 6, which had been surface sterilized. Ten seeds were cultivated per pot, three pots were used per replicate and each treatment consisted of three replicates. After 10, 20, and 40 days of planting, root and crown rot severity percentages were assayed.

#### 2.3.5. Disease Assessment

To assess the disease severity, a modified disease rating scale from 0 to 4 was used as follows: 0 = healthy (no visible lesion), 1 = 0.1–2 cm lesion length on stem, 2 = 2.1–3 cm lesion length on stem, 3 = 4.1–6 cm lesion length on stem, 4 = ≥ 6.1 cm lesion length on stem or dead plant [20].

The disease severity was determined by the length of the lesion on the infected stem [21]. The infected area was determined from the plants in each pot, and the mean was obtained for each treatment. The disease severity was calculated using the formula of Wheeler, [22]:

Disease severity = Sum of individual ratings/(No. of plants observed x Maximum disease rating) × 100
(1)

### 2.4. In Vivo Effects of AgNs Foliar Spraying on Sunflower Plants Infected by S. rolfsii

This research was conducted in a greenhouse located at Plant Pathol. Dept., Fac. Agric., Minia University, EL-Minia, Egypt.

It evaluated the effect of AgNs at concentrations of 25, 50 and 100 µg/mL in preventing the infection of the root and collar rot incited by *S. rolfsii* on sunflower plants cv. Sagha 53. A total of 100 seeds were sown in five replicate pots for each treatment (5 seeds per pot). *S. rolfsii* was grown on autoclaved barley grains (100 g and 65 mL water per flask). The inoculation was performed using 5 mm diameter agar discs. The flasks were incubated at 28 °C for 10 days to reach sufficient fungal growth, then mixed with soil at 2.5 percent w/w and placed into 15 cm diameter pots. The control was sterile soil that was not infected with *S. rolfsii*. The seedlings at age of 3 weeks were sprayed with AgNs solution at different concentrations and then sprayed again after 3 weeks. After 24 h, the soil moisture was corrected to 50% of its water holding capacity, and the amount of water loss was recovered [23].

Disease Assessment

The arbitrary (0–5) disease scale described by [24] was used to measure the disease severity, in which: 0 = no infection, 1 = 1–20% infected plants; 2 = 21–40% infected plants; 3 = 41–60% infected plants; 4 = 61–80% infected plants; 5 = 81–100% infection. The disease severity was determined using the method below.

Disease severity = 0A + 1B + 2C + 3D + 4E + 5F/5P × 100
(2)

where A, B, C, D, E, and F is the number of plants in each disease severity class and 5P refers to the total number of plants (T) multiplied by the highest disease grade 5 [20].

### 2.5. Statistical Analysis

SAS 2013 software (SAS Institute, Cary, NC, USA) and analysis of variance have been used to analyze the data (ANOVA). To examine for significant differences between the main treatments, a general linear model (GLM) approach was applied. Tukey’s method was used to compare the means (*p* < 0.05).

## 3. Results

### 3.1. Biosynthesis and Characterization of AgNs

**Figure 1:**
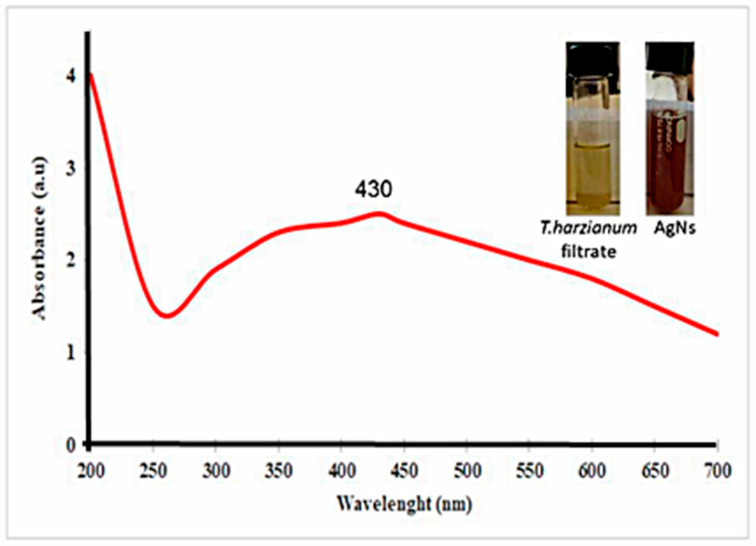
The change of the color and UV-Vis spectrum of synthesized AgNs at the wavelength range from 200 to 700 nm.

**Figure 2:**
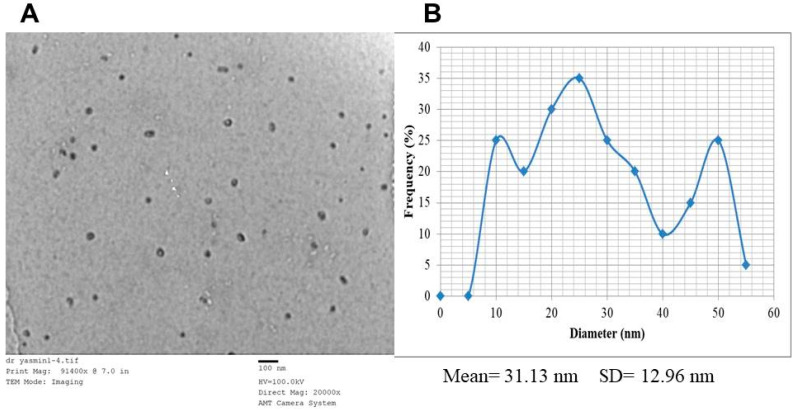
Characterization of AgNs synthesized by *T. harzianum* using TEM analysis at 91,400× (**A**), and the size particle distributions depend on the TEM image (**B**).

**Figure 3:**
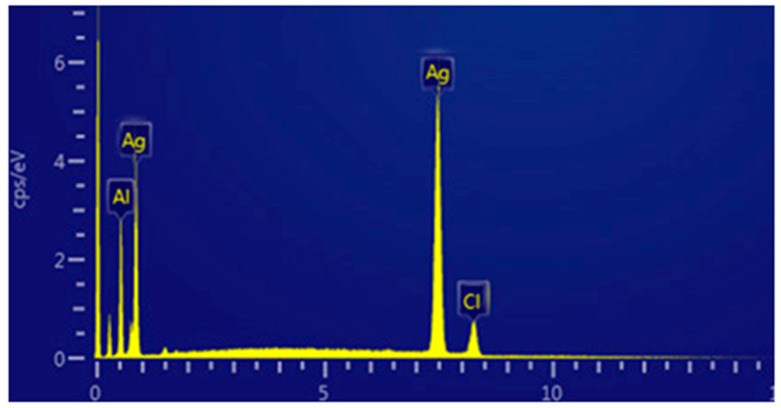
The EDS spectra of prepared AgNs.

The appearance of brown color indicated the biogenesis of nanoparticles. The UV-Vis absorption spectra of AgNs suspension observed at 430 nm are presented in (Figure 1). The TEM images indicated that the biosynthesized AgNs have a spherical shape with an average particle size of 31.13 nm as shown in Figure 2A,B. The energy dispersion spectrum (EDS) analysis indicated that obtained nanoparticles contained Ag 68.8, Al 22.1 and 9.1% Cl (Figure 3).

### 3.2. Antifungal Activity of AgNs

#### 3.2.1. In Vitro Effect of AgNs on the Growth of Sclerotinia sclerotiorum and Sclerotium rolfsii

**Figure 4:**
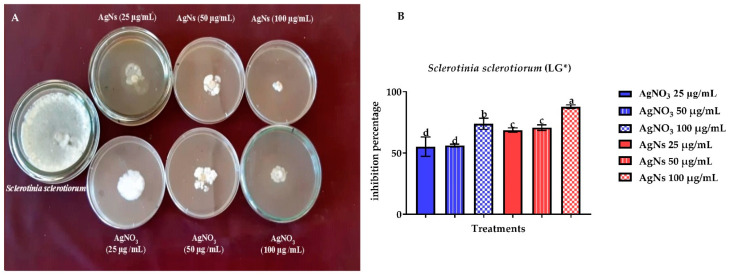
The in vitro inhibitory effect of AgNs and AgNO_3_ on the linear growth of *Sclerotinia sclerotiorum* (**A**,**B**). ^a–d^ Column with different superscripts significantly differs at *p* < 0.05. * Linear growth.

**Figure 5:**
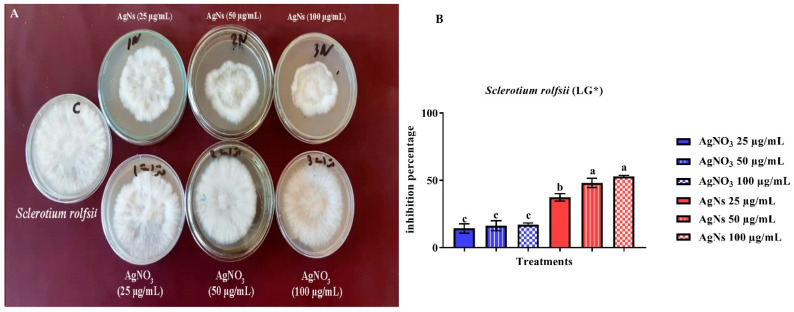
The in vitro inhibitory effect of AgNs and AgNO_3_ on the linear growth of *Sclerotium rolfsii* (**A**,**B**). ^a–c^ Column with different superscripts significantly differs at *p* < 0.05. * Linear growth.

The current study found that bulk Ag effectively suppressed the development of *S. sclerotiorum* and *S. rolfsii* on a nutrient agar (NA) medium. However, a greater effect was shown by nanoparticles (Figure 4). AgNs at the final concentrations of 25, 50 and 100 μg/mL showed a mean inhibitory percentage *S. sclerotiorum* growth of 68.7, 70.7, and 87.8% compared with that of silver nitrate that presented a mean inhibitory activity of 55.2, 56.1, and 73.9%, respectively. The AgNs inhibition of linear growth of *S. rolfsii* was 52.8, 48.0, and 37.3% and for AgNO_3_ was 17.1, 16.2 and 14.3%, respectively (Figure 5).

#### 3.2.2. Mycelial Dry Weight (DW)

**Figure 6:**
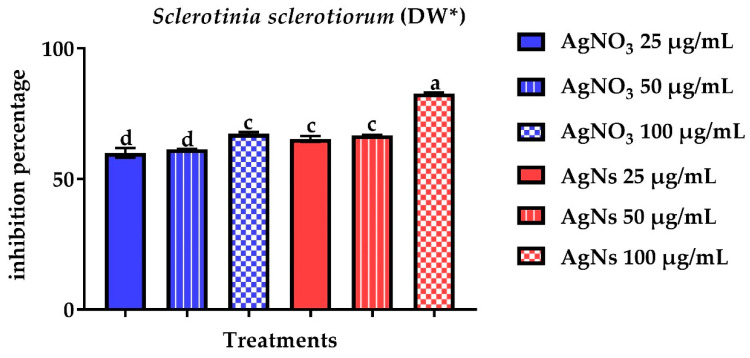
Effect of AgNO_3_ and AgNs on the mycelial dry weight of *S. sclerotiorum*. ^a–d^ Column with different superscripts significantly differs at *p* < 0.05. * Dry weight.

**Figure 7:**
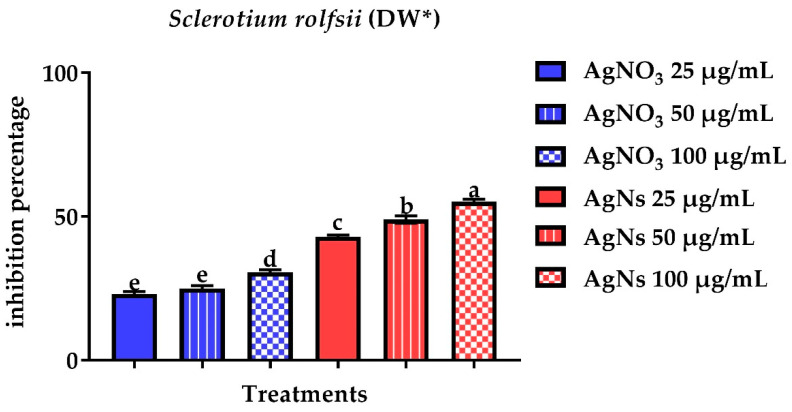
Effect of AgNO_3_ and AgNs on the mycelial dry weight of *Sclerotium rolfsii.* ^a–e^ Column with different superscripts significantly differs at *p* < 0.05. * Dry weight.

To determine antifungal activity, the pathogenic fungi’s mycelial dry weight was measured. There was a significant reduction in mycelial dry weight since a considerable inhibition of 82.7% in DW for *S. sclerotiorum* (Figure 6) and 55.1% for *S. rolfsii* was found at 100 µg/mL concentration (Figure 7).

#### 3.2.3. Number of Sclerotia

**Figure 8:**
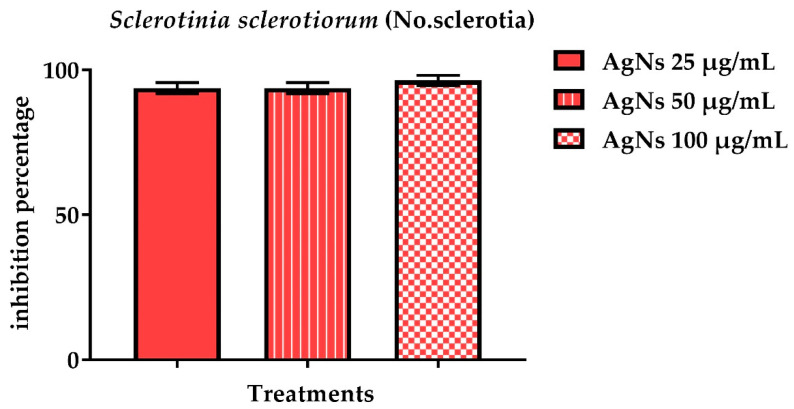
Effect of AgNO_3_ and AgNs on the number of sclerotia of *Sclerotium rolfsii*.

**Figure 9:**
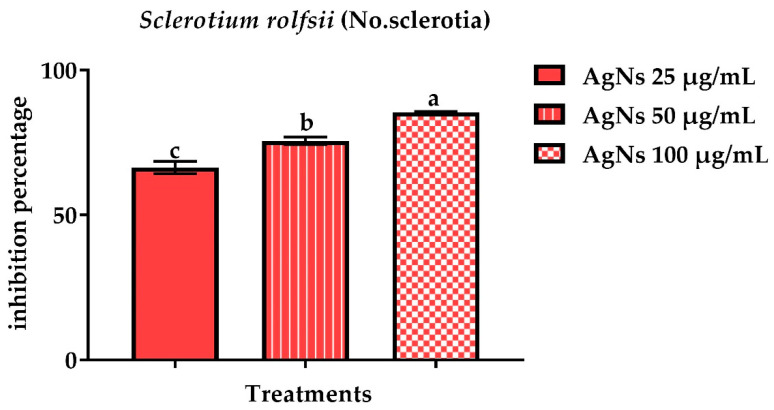
Effect of AgNO_3_ and AgNs on the number of sclerotia on *S. rolfsii.* ^a–c^ Column with different superscripts significantly differs at *p* < 0.05.

The number of sclerotia was used to calculate the inhibitory activity of AgNs in NB broth. Figure 8 and Figure 9 show that all the AgNs concentrations tested considerably reduced the quantity of *S. sclerotiorum* sclerotia as compared to the control. The AgNs at100 µg/mL concentrations were the most successful, with a percentage of inhibition of a number of sclerotia of 96.4%, while *S. rolfsii* had a lower percentage of inhibition of 85.4%.

#### 3.2.4. In Vivo Effect of AgNs on the Severity of Root Rot Disease

**Figure 10:**
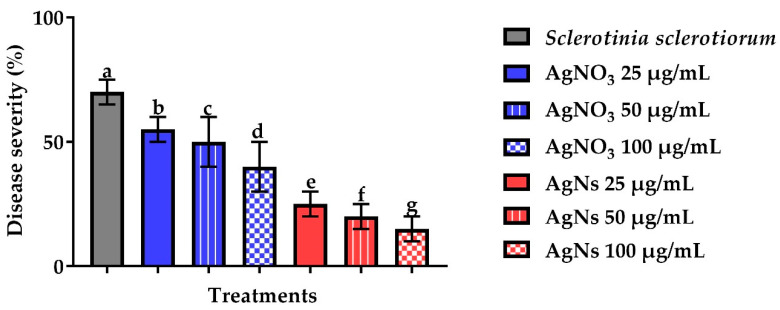
Effect of AgNO_3_ and AgNs on the incidence and severity of root rot disease of bean plants. ^a–g^ Column with different superscripts significantly differs at *p* < 0.05.

**Figure 11:**
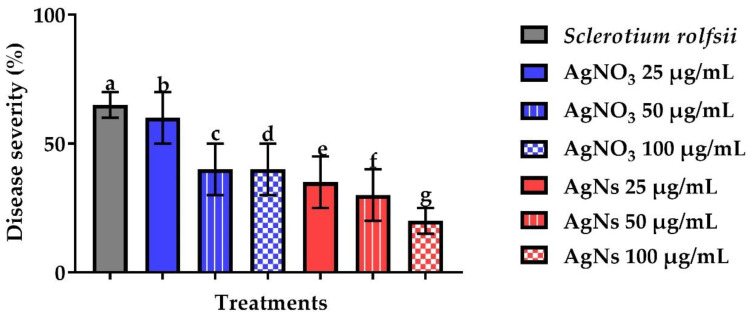
Effect of AgNO_3_ and AgNs on the incidence and severity of root rot disease of sunflowers plants. ^a–g^ Column with different superscripts significantly differs at *p* < 0.05.

Data presented in (Figure 10) showed the effect of different concentrations of AgNs on disease severity in bean plants inoculated with *S. sclertiorum.* The disease severity significantly decreased with the three tested concentrations, however, the most effective was at 100 µg/mL which was 15% if compared to 70% for the infected bean plants. On the other hand, sunflowers plants infected with *S. rolfsii* (Figure 11) showed a reduction in severity from 20% to 65% for the infected control at a concentration of 100 µg/mL.

## 4. Discussion

The use of fungi covers a large area in the synthesis of metal nanoparticles owing to their easy handling and cultivation, high biomass production and the secretion of large quantities of metabolites, enzymes, and extracellular proteins, thus making them promising materials for use in the areas of health, agriculture, and the environment [25]. Silver nanoparticles have unique optical, electrical, and thermal characteristics that make them ideal for many biological applications [5]. Silver nanoparticles could adhere to the cell walls and membranes of microorganisms and then may get inside the cells, impair the cell structure, induce the production of reactive oxygen species, and disrupt the signal pathway [26]. These properties make AgNs promising agents for the control of pathogens in agriculture and human and animal health [23].

Due to the presence and spread of bacteria resistant to various antibiotics, silver-based antiseptics have received increased attention in recent years. The fungus *Trichoderma viride* was used to biosynthesize silver nanoparticles by using aqueous silver (Ag^+^) ions that were exposed to a *T. viride* filtrate, which were then reduced in solution, resulting in the creation of very stable AgNs with sizes ranging from 5 to 40 nm [27]. It is important to note that AgNs are generally employed for plant disease control as a result of their suppressive action against a wide spectrum of diverse plant diseases [28]. We discovered that synthesized AgNs had a good inhibitory action against hyphal development, sclerotia production, and myceliogenic germination of sclerotia, indicating their potential use in antifungal therapy. Furthermore, as we could not purify the synthesized AgNs in our study, the inhibitory effects could have resulted from the AgNs and *T. harzianum* metabolites, which may have had a synergetic effect. Kim et al. [28] examined the antifungal action of AgNs against eighteen phytopathogenic fungi and the most significant suppression of phytopathogenic fungi was at 100 ppm, which agrees with our results. The interaction of AgNs with fungi induces several changes in the cell wall structure of the fungus, including AgNs contact, accumulation, lamellar fragments, and the creation of micropores or fissures, finally allowing AgNs to enter the cell. A similar study discovered that nanoparticles’ antifungal activity emerged from the first direct contact with fungal cell walls, generating ROS formation, affecting membrane integrity, and changing morphological features [29]. The AgNs coated with fungi-derived capping has been shown to possess great biological activity [9,30]. To protect plants against disease invasion, nanoparticles are applied to seeds or leaves in the soil. As a result, the NPs may be able to control infections in a similar way as chemical pesticides. AgNs have been used in plant disease management instead of using chemical fungicides [31,32,33]. Lamsal et al. [32] found that cucumber plants treated with AgNs showed the lowest disease incidence % at a concentration of 100 ppm. As a result, the goal of this work was to get a better knowledge of the antifungal mechanism of nanoparticles, which might possibly be effective in the prevention of various fungal diseases. The use of AgNs in the form of nano pesticides in agroecosystems has not yet been thoroughly investigated, and future research should concentrate on analyzing potential hazards to achieve safer and more efficient agricultural practices [34].

## 5. Conclusions

In conclusion, nanotechnology is considered an innovation in agriculture as an alternative tool to harmful pesticides. This study investigated whether it is possible to biosynthesize silver nanoparticles using *Trichoderma harzianum*, which acts as a reducing agent. The formation of AgNs was confirmed by UV spectroscopy. The biosynthesized AgNs showed an inhibitory effect on *S. sclerotiorum* and *S. rolfsii* growth. The synthesized nanoparticles possess a great capacity of suppressing *S. sclerotiorum* and *S. rolfsii* infections in bean and sunflower plants, respectively. Although we were successful in biosynthesizing AgNs, further research is needed to properly use fungus for biogenic synthesis, such as understanding the processes of fungal metabolites that may have biological activity and function in synergy with the nanoparticle.

## Data Availability

The data presented in this study are available on request from the corresponding author.
